# Virtual Schooling in Dental Undergraduate and Postgraduate Education: A Knowledge, Attitude, and Practice (KAP) Survey

**DOI:** 10.7759/cureus.49850

**Published:** 2023-12-02

**Authors:** Lichi Solanki, Swapna Sreenivasagan, Seerab Husain, Shweta Nagesh

**Affiliations:** 1 Orthodontics and Dentofacial Orthopaedics, Saveetha Dental College and Hospitals, Saveetha Institute of Medical and Technical Sciences, Saveetha University, Chennai, IND

**Keywords:** dental students, access to education, virtual learning, dental, dental education

## Abstract

Aim

The purpose of this survey was to appraise the knowledge, attitude, and practice (KAP) of dental undergraduate and postgraduate students in India towards virtual learning and provide an improved understanding of practicing this learning strategy as a complementary tool for the evolution of dental education.

Materials and methods

A cross-sectional, closed-ended, multiple-choice online questionnaire was distributed on a national scale to undergraduate and postgraduate dental students throughout India. The questions focused on the choice of virtual platforms, learning methods, duration of classes, mental health, pros and cons, and approach to virtual education as a complementary tool in the future. The survey was distributed in the form of a web-based link and quick-response (QR) code via various social platforms. Descriptive statistics was performed to compile the data.

Results

A total of 2130 students (1318 undergraduate and 812 postgraduate students) participated in the study. The study found that 81.1% of the undergraduate and 92.5% of the postgraduate students were exposed to virtual learning. Zoom and Google Meet were the most widely used platforms for online education. The most common mode of virtual learning was webinars conducted by subject faculties. Ease, comfort of access, and flexibility of time were the most common advantages, while internet connectivity issues along with negative psychological impact are a few common drawbacks. The concept of blended learning was most preferred by the students.

Conclusion

Undergraduate and postgraduate students showed good knowledge and a positive attitude to virtual schooling and preferred a hybrid model of instruction.

## Introduction

The coronavirus disease 2019 (COVID-19) pandemic forced lockdowns everywhere, and the medical fraternity advised everyone to maintain social distancing to prevent the spread of disease. This resulted in disturbances in many areas of society, which had an effect on the educational sector. In order to give education without interruption and support the academic growth of dental students, a change was required in the method of education [1]. The traditional face-to-face learning was accommodated to virtual learning methods in some areas of dentistry [2]. The expeditious advancement of computer technologies and growth in internet base over the years led to the rapid development in online education. The expanded access to computers, faster network connectivity, and technologies has increased the adoption in various fields, and dental education is no exception. Social media platforms offering educational tools through smart devices in the form of apps like Facebook, YouTube, Twitter, Zoom, Instagram, Skype, etc. have resulted in increased collaborations, connections, and communications on a global scale in the younger generation [3-6]. This early adoption by the younger generation paved the way into the field of learning through virtual education. Virtual learning is comprised mainly of online seminars, demonstration videos, case scenarios, and discussions on social media platforms like Zoom, Google Meet, Google Classroom, Skype, etc. [7,8].

Previous literature studies by Barry et al. suggested that the academic anatomy community may find value in the integration of social media into blended learning approaches in anatomy programs. This suggested that a vast majority of the learners preferred electronic (e)-learning over traditional classroom learning owing to ease of accessibility, time constraints, ease of understanding, and more pictorial representations [9]. This mode of learning was also adopted by students in medical and dental education to keep up with increased loads of new and updated topics [10]. Although there were many advantages, there were drawbacks as well, including difficulties like availability of technology with all students and universities, maintaining attention in front of a computer screen for extended periods of time, students getting easily distracted, improper interpersonal communication with peers and teachers, and consequences on mental health [11-14].

Virtual learning alone may not suffice for imparting overall dental education. But incorporating virtual learning with traditional classroom learning could lead to enhanced student learning. Understanding the pros and cons, obstacles, and challenges of virtual education in comparison with traditional learning from an undergraduate and postgraduate student's perception would help in forming a structured protocol for future dental education. Thus, the aim of this study was to conduct a nationwide survey to assess the knowledge, attitude, and perception (KAP) of undergraduate and postgraduate dental students towards virtual learning as a complementary tool in dental education and their approach towards dental virtual education system.

## Materials and methods

The study was conducted at Saveetha Dental College and Hospitals, Chennai, India, from May 6 to December 6, 2020, and received approval from the Institutional Review Board (IRB) of the said institution (approval number: IHEC/SDC/ORTHO-1908/21/202).

Selection and description of participants

A cross-sectional online survey was conducted nationwide directed towards the undergraduate dental students all across India. The survey recruited all the undergraduate, intern, and postgraduate dental students. 

Questionnaire

The questionnaire consisted of closed-ended multiple-choice questions that was formulated using a smart survey online application and can be found in the Appendices section. All the data was stored safely in the sub-processors. The questionnaire used in the study was developed and validated based on a pilot study. A preliminary pilot survey was conducted on 20 undergraduate and 20 postgraduate students who were exposed to virtual learning. An email with a link along with a quick-response (QR) code through WhatsApp was used for the distribution of the pilot survey. The validation of the questionnaire was done, and the results of the feedback were taken into consideration in designing the final questionnaire. The survey consisted of 22 closed-ended multiple-choice questions for undergraduates and 19 for postgraduates. The basic demographic data of the students and year of their education were recorded. The formulated questions were directed on choice of social platforms, inclination of learning methods, advantages and disadvantages of virtual learning, duration of online classes, mental health of students, and their perception of virtual education as a complementary tool in future dental education. The questionnaire was formulated in such a way that students having no exposure to virtual learning were not compelled to answer all the questions and were directed towards the end where their impression regarding virtual education was recorded.

Prior consent was obtained from all the participants, and participants who did not wish to consent were directed towards the end of the survey. The participants were not subjected to personal questions, and an email with a link describing the intent of the survey was distributed among the students. The validated questionnaire was used for the final survey. The final validated survey which was created using the hypertext markup language (HTML) was made available to potential respondents via a web server. The link was shared across platforms like email, WhatsApp, Instagram, and Facebook to all potential undergraduate and postgraduate students across the nation. It was distributed in professional groups as personal mails and messages. The exclusion criteria consisted of the students who participated in the pilot survey and the members of the questionnaire design team. Three reminder messages were sent across a span of one week. The survey link was disabled after the last reminder message in order to avoid duplicate responses. The Internet protocol (IP) addresses were collected, and the anonymity of the survey was guaranteed as the investigators had no means of identifying the participants.

Statistical analysis

All the collected data were analyzed using the IBM SPSS Statistics for Windows, Version 23.0 (Released 2015; IBM Corp., Armonk, New York, United States). Descriptive statistics were used to present the results of the survey.

## Results

A total of 2130 students (1318 undergraduate and 812 postgraduate students) responded to the survey, of which 16 participants did not complete the survey. The survey had an overwhelming 99% response rate. A total of 1302 participants (74.5% females; 25.5% males) were undergraduate students, and 812 participants (73.2% females; 26.8% males) were postgraduate students. In the present study, 92.5% of the postgraduate students and 81.1% of the undergraduate students had been exposed to virtual learning methods. Students who denied having been exposed to any form of online learning were directed to more general questions and were not subjected to virtual learning-related questions.

Undergraduate students

Undergraduate students in their second year made up the majority of the sample (29.1%). Students in all other years were equally represented. The vast majority of the undergraduate students (64.1%) used mobile phones for their web-based learning through different platforms. A significant percentage of students used the Zoom platform (78.22%) for university dental education followed by Google Meet (30.21%) and the WhatsApp video platform (16.57%). Publicly available video/audio communication platforms were used by 29.9% of the students, while students enrolled in institutions presenting concerns regarding security and privacy (24.3%) used secured private institutional platforms. The classes were conducted for a period of six days a week for 45.74% of the students, and others had classes three to five days a week. Online seminars/lectures by faculty were the commonest mode of learning, and the most preferred duration was 45-60 minutes. Virtual education as a teaching method employed in institutions comprised webinars or lectures (72%), assignments (54.5%), and online tests/exams (43.4%).

The common modes of learning opted by students were webinars (60%), clinical demonstrations (55.8%), case scenarios (44.8%), activity learning (44.3%), and group discussions (43.5%). Activity-based learning methods like flowcharts (69.5%), mind maps (50.7%), and quizzing (49%) were the preferred choice by many students. The interaction between students and faculty was rated as either the same (34.5%) or better (33.7%) by majority of the students; only 21.9% of the students thought the interaction was worse. The idea of conventional classroom learning was chosen by 48% of the respondents, and only 6.54% supported virtual learning alone, whereas 45.5% of the students found a combination of the two learning methods to be beneficial. The major advantages and disadvantages of the virtual education are depicted in Table 1 and Table 2. 

**Table 1:** Survey questions and responses pertaining to the advantages of virtual schooling

S. no.	Advantages	Response
1	Flexibility of time	60.45%
2	Ease of access (using computer or smartphone)	55.15%
3	Access from any location	65.05%
4	Affordable	42.70%
5	Access to faculty without any limitation	30.65%
6	Multidimensional possibility to enhance knowledge through online sources	29.03%
7	Ease of assessment	29.26%
8	Environment-friendly	30.80%
9	Faster access to and sharing of educational resources	44.24%
10	Others (please specify)	1.54%

**Table 2:** Survey questions and responses pertaining to the disadvantages of virtual schooling

S. no.	Disadvantages	Response
1	Internet connectivity issues	88.16%
2	Cost of internet connection	31.07%
3	Cost of devices	21.58%
4	Internet security issues	52.03%
5	Lack of face-to-face interaction with faculty	53.76%
6	Lack of interaction with patients	65.23%
7	Lack of interaction with classmates	65.23%
8	Laziness and procrastination	44.88%
9	Need for self-discipline	29.22%
10	Poor pre-clinical and practical orientation	45.99%
11	Feeling of isolation, lack of social contact	17.02%
12	Impact on physical and mental health	45.50%
13	Others (please specify)	0.74%

The major advantages that were brought along with virtual learning were comfort of access from any location (65.05%) and flexibility of time (60.1%) followed by ease of accessibility (55.2%). The most common obstacle encountered in virtual education were the internet connectivity issues (85.7%) followed by lack of patient interaction (56.91%). The other common difficulties which the students came across were internet security issues (41.6%) and negative impact on health (49.6%). 50.4% of the survey participants believed that virtual learning compromised clinical understanding. The notion of implementing it in future dental education was not supported by 50.1% of the students. A significant number (43.6%) of students believed it had a negative impact on overall health, whereas 72.9% agreed to its usefulness in the pandemic times.

Postgraduate students

The survey included first, second, and third year postgraduate students from all branches of dentistry, with 33.1% being orthodontists. The greater number of postgraduate students used laptops for attending online classes (65.5%), mobile phones (26.3%), tablets (5.6%), and desktop computers (2.7%). The modes of education offered by web-based applications used for learning were Zoom application (85.7%), followed by Google Meet (29.2%), Cisco Webex (26.4%), GoTo Webinar (22%), and Facebook Live (17.5%). The duration of the virtual learning through webinars offered by faculty was 60 minutes and journal reading 45 minutes. Various other types of virtual learning have been tabulated in Table 3. The classes were conducted for a period of six days a week for 33.3% of the students, while others had classes three to five days a week. The interaction with faculty in virtual sessions was the same as classroom sessions as stated by 46.3% of the students, and 33.2% of the students believed the interaction was better, while a small percentage of students (20.53%) felt the interaction was much worse. A greater majority of 58.8% of the students were of the opinion that both virtual and classroom learning were beneficial to them, whereas 35.6% of the postgraduates still supported the idea of classroom learning.

**Table 3:** Type of virtual learning and its duration for postgraduate students

Type of virtual learning	Duration of the session							
	Not used	15 min	30 min	45 min	60 min	90 min	120 min	>120 min
Webinars conducted by faculty (lectures/seminars)	12.7% (95)	0.5% (4)	6.5% (49)	24.4% (183)	31.5% (236)	14.3% (107)	4.9% (37)	5.2% (39)
Webinars conducted by students	11.3% (85)	1.6% (12)	11.6% (87)	28.0% (210)	29.6% (222)	11.9% (89)	3.6% (27)	2.4% (18)
Journal clubs	12.1% (91)	3.2% (24)	28.0% (210)	29.3% (220)	17.9% (134)	6.7% (50)	2.0% (15)	0.8% (6)
Clinical demonstrations	60.7% (455)	4.0% (30)	12.7% (95)	11.6% (87)	6.9% (52)	2.7% (20)	0.5% (4)	0.9% (7)
Non-clinical demonstrations	59.2% (444)	6.3% (47)	13.1% (98)	11.5% (86)	6.8% (51)	2.1% (16)	0.4% (3)	0.7% (5)
Case scenarios/discussions	37.9% (284)	5.2% (39)	18.3% (137)	19.5% (146)	12.3% (92)	4.7% (35)	1.1% (8)	1.2% (9)
Activity-based learning	60.3% (452)	3.7% (28)	11.9% (89)	10.7% (80)	8.3% (62)	2.5% (19)	0.9% (7)	1.7% (13)
Group discussions	33.5% (251)	8.9% (67)	19.3% (145)	16.1% (121)	14.5% (109)	5.2% (39)	1.2% (9)	1.2% (9)
Assignments	54.8% (411)	4.4% (33)	13.5% (101)	10.1% (76)	8.4% (63)	2.8% (21)	1.3% (10)	4.7% (35)
Self-learning through videos	23.1% (173)	8.1% (61)	23.1% (173)	14.0% (105)	17.5% (131)	6.0% (45)	2.7% (20)	5.6% (42)
Online tests/exams	51.1% (383)	2.9% (22)	8.8% (66)	4.7% (35)	10.8% (81)	3.5% (26)	5.1% (38)	13.2% (99)
Others (please specify)	89.1% (668)	1.1% (8)	3.2% (24)	3.2% (24)	2.1% (16)	0.4% (3)	0.3% (2)	0.7% (5)

Comfort of access from any location was the greatest advantage of this educational pattern according to 72.9% of the postgraduates. Other advantages that were among the top few were flexibility of time (62.8%), ease of accessibility (58.8%), faster sharing of resources (56.5%), and affordability (45.7%). In this study, 88.16% of the students quoted internet connectivity issues as a challenge in continued online education. The other disadvantages that were sought by the students in high numbers were lack of interaction with patients (65.23%) and faculty (53.76%).

The idea of conducting conferences on virtual platforms was supported by 47.5% of the postgraduates. Paper presentations (58%) and poster presentations (62%) on virtual platforms were also opted by a vast number. A higher majority of 89.5% of the respondents were of the opinion that virtualizing education during the lockdown period was very helpful. Around 47.5% of the students agreed that using virtual platforms as a supplement to traditional methods as opposed to using it as a major mode of education was a good strategy.

## Discussion

The survey was aimed at assessing the perception and knowledge of the undergraduate and postgraduate students about virtual learning as an adjunct to conventional methods used in dental education. A study by Hong et al. [15] found that 95.7% of the students considered e-learning as compelling, which was similar to the present study. The questionnaire designed has highlighted the various aspects of virtual learning from the student's perspective wherein the majority of the participants were females (74.5% undergraduates, 73.2% postgraduates), due to the high number of females pursuing dental education [16].

The bulk of the students reported using Zoom, WhatsApp video call, Google Meet, and YouTube Live, and a staggering 81.1% of the undergraduates and 92.5% of the postgraduates used these platforms for educational purposes which is similar to a previous study [17]. In the present study, the majority of the students selected the Zoom option followed by Google Meet and WhatsApp video call for online education, and a minority of students used other platforms.

A high number of undergraduates utilized mobile phones for virtual courses, whereas fewer than one-third of undergraduate students reported using tablets or laptops, and the least number of students reported using a desktop computer for studying, which are consistent with results of the other studies [15,18]. The attitude of students towards using mobile phones as a medium of providing dental education was quite favourable due to availability and accessibility to economic phones and mobile data packs [19]. About 24.5% of the students preferred secure video conferencing platforms as a mode of dental education which is similar to a study by Debnath et al. [20]. Also, a study by Lin et al. [21] found high usage and preference of smartphones among students which is similar to the present study. This also entails future dental educators to design contents which are also compatible with mobile devices. Among the activity-based learning, mind mapping, flowcharts, and quizzing topped their lists. Quizzing and assignments were a priority of students similar to the present study [22].

Duration of webinars conducted by faculty for undergraduates were 45-60 minutes, and this was also the preferred choice by the students. According to the study by Schonwetter et al. [22], the number and duration of online classes, as well as the number of students enrolled, grew substantially. The interaction between students and faculty was quite low where 35.5% of the undergraduates and 33.2% of the postgraduates were convinced and the rest of them felt classroom learning had better interaction among peers and faculty. Blended learning was favoured by 45.55% of the undergraduate students and 58.80% of the postgraduate students. These results were concurrent with the results of the study done by Puryer et al. [18]. In terms of increasing clinical skills, e-learning was deemed less effective than face-to-face learning in medical fraternities as mentioned in the study by Shrivastava et al. [23] which was similar to the present study.

The fundamental challenges that the students encountered are the internet connectivity issues (85.79%). Not having a stable internet connection is one of the biggest problems. Higher bandwidth connections are not available or not affordable to all students. This was one of the most common difficulties in virtual schooling. Along with connectivity problems, internet security issues were also a concern for 48.54% of the responders which is similar to other studies [20,24]. Lack of interaction with faculties, patients, and fellow classmates is one of the primary issues according to the students. As there is no direct clinical evaluation of the patients, this could affect and compromise the clinical understanding of the topic (50.4%). Another set of negative aspects of virtual learning that the students agreed upon were the negative attitude, procrastination (44.9%), which affects their self-discipline (29.2%), and a negative impact on overall physical and mental health (45.5%) resulting in efficiency. While trying to assess the relation between mental health and virtual learning, 50% of the students had no ill effect on mental health, while an equal percentage of students mentioned problems with mental health. Students claimed to have faced physical and mental disorientations affecting their overall health and education. Studies have similar results stating that online learning has a negative impact on mental health [15,25,26].

Limitations

The questionnaire was tedious due to the large number of questions. Another limitation was its restriction solely to the Indian population. Thus, conducting a multinational survey would allow us to gain a global perspective on virtual dental education. The questionnaire was performed during pandemic periods, which may have influenced the results obtained, contributing to another limitation of the study.

## Conclusions

The undergraduate and postgraduate students had good knowledge about the various platforms used in dental education and their advantages and disadvantages. They also showed a positive attitude towards virtual schooling. However, with regard to the practice of this method in dentistry, both undergraduates and postgraduates preferred a combination of virtual and traditional method of instruction.

## Appendices

Undergraduate survey questions

All the questions were added to Google Docs.

1. Clicking on the "AGREE" button below indicates that I have read the above information and I agree to participate. Are you interested in the survey?

I AGREE to participate

I do not want to participate

2. Sex

3. Name the state/union territory you are studying in?

4. In which dental school are you currently studying?

5. In which year are you studying?

First year BDS

Second year BDS

Third year BDS

Fourth year BDS

Internship

6. Which kind of video platform have you used for the purpose of non-dental education purposes before? (Multiple responses allowed)

Zoom

Skype

Facebook Live

Instagram Live

YouTube Live

GoTo Webinar

Google Meet

Google Hangouts

Microsoft Teams

Cisco Webex

GoTo Meeting

TechSmith

WhatsApp video call

Livestorm

Join.me

None of the above

Others (please specify):

7. Virtual learning is a learning experience that is enhanced through utilizing computers and/or the internet both outside and inside the facilities of the educational organization. The instruction most commonly takes place in an online environment. Have you been exposed to any form of virtual learning in dentistry?

Yes/no

8. Which type of device do you mostly use for virtual learning?

Desktop

Laptop

Tablet

Mobile phone

9. Which kind of video platform have you used for university dental education: lectures, case discussions, and seminars? (Multiple answers allowed)

Zoom

Skype

Facebook Live

Instagram Live

YouTube Live

GoTo Webinar

Google Meet

Google Hangouts

Microsoft Teams

Cisco Webex

GoTo Meeting

TechSmith

WhatsApp video call

Livestorm

Join.me

Others (please specify):

10. In terms of security, which type of video platform do you regularly use for online dental lectures?

Institutional secure video conferencing platforms (by the university, government, etc.)

Common access video communication platforms

Both of the above

I don't know

11. Which type of virtual learning in dentistry do you use in your university? (Multiple responses allowed)

Webinars conducted by faculty (lectures/seminars)

Webinars conducted by students

Clinical demonstrations

Non-clinical demonstrations

Case scenarios/discussions

Activity-based learning

Group discussions

Assignments

Self-learning through videos

Online tests/exams

None of the above

Others (please specify):

12. Which type of virtual learning in dentistry would you prefer? (Multiple responses allowed)

Webinars conducted by faculty (lectures/seminars)

Webinars conducted by students

Clinical demonstrations

Non-clinical demonstrations

Case scenarios/discussions

Activity-based learning

Group discussions

Assignments

Self-learning through videos

Online tests/exams

None of the above

Others (please specify):

13. Which activity-based learning do you think is effective for virtual learning in dentistry? (Multiple responses allowed)

Mind maps

Flowcharts

E-posters

Quizzing

Crosswords

Puzzles

Fill ups

Match the following

None of the above

Others (please specify):

14. What is the average duration of one virtual theory class in your university?

15 minutes

30 minutes

45 minutes

60 minutes

120 minutes

>120 minutes

15. What is the frequency of virtual classes you are attending per week in your university?

One day a week

Two days a week

Three days a week

Four days a week

Five days a week

Six days a week

Seven days a week

Others (please specify):

16. What is the frequency of virtual classes you would prefer to attend at your university?

One day a week

Two days a week

Three days a week

Four days a week

Five days a week

Six days a week

Seven days a week

Others (please specify):

17. How do you rate the interaction between you and your faculty using virtual learning as compared to classroom learning?

The interaction is better

The interaction is worse

The interaction is the same

18. Did you experience a negative impact on virtual learning in dentistry due to a negative effect on mental health and well-being caused by the lockdown?

Yes/no

19. Which mode of learning in dentistry do you think is more beneficial for you?

Virtual learning

Classroom learning

Both

20. What do you think are the advantages of virtual learning in dentistry? (Multiple responses allowed)

Flexibility of time (can be viewed at a convenient time for the student)

Ease of accessibility (using a computer, smartphone, or tablet)

Comfort of access from any location (no need to be in the classroom)

Affordable (eliminates travel costs and travel time)

Remote access to faculty without geographic limitations

Multidimensional possibility to enhance knowledge through online resources

Ease of assessment (assigned tasks, knowledge, clinical competence)

Environment-friendly

Faster access and sharing of educational resources (reading materials, presentations, e-books, videos)

Others (please specify):

21. What do you think are the disadvantages of virtual learning in dentistry? (Multiple responses allowed)

Internet connectivity problems

Cost of internet connections

Cost of devices (computer, tablet)

Internet security issues (data, sensitive patient information)

Requirement of basic computer skills

Lack of face-to-face interaction with the faculty

Lack of interaction with patients

Lack of interaction with classmates

Students' weak attitude (laziness, procrastination, etc.)

Need of self-discipline

Need of self-organization

Poor pre-clinical/clinical/practical orientation

Feeling of isolation

Negative impact on physical and mental health (strain to the eyes, sedentary lifestyle, etc.)

Others (please specify):

22. I think virtual learning in dentistry ______________________

Postgraduate survey questions

All the questions were added to Google Docs.

1. I am a/an

Undergraduate student

Postgraduate student

Faculty

Dental practitioner/specialist

I am not a dentist or a dental student

2. Clicking on the "AGREE" button below indicates that I have read the above information and I agree to participate. Are you interested in the survey?

I AGREE to participate

I do not want to participate

3. Sex

4. Name the state/union territory you are studying in?

5. In which dental school are you currently studying?

6. Which branch of dentistry are you studying in?

Orthodontics

Periodontics

Prosthodontics

Oral and maxillofacial surgery

Oral medicine and radiology

Pedodontics

Endodontics

Oral pathology

Public health dentistry

7. In which year are you studying?

First year MDS

Second year MDS

Third year MDS

8. Virtual learning is a learning experience that is enhanced through utilizing computers and/or the internet both outside and inside the facilities of the educational organization. The instruction most commonly takes place in an online environment. Have you been exposed to any form of virtual learning in dentistry?

Yes/no

9. Which type of device do you mostly use for virtual learning?

Desktop

Laptop

Tablet

Mobile phone

10. Which kind of video platform have you used for university dental education: lectures, case discussions, seminars, and journal clubs? (Multiple answers allowed)

Zoom

Skype

Facebook Live

Instagram Live

Google Hangouts

Microsoft Teams

Cisco Webex

Google Meet

GoTo Webinar

GoTo Meeting

WhatsApp video call

YouTube Live

None of the above

Others (please specify):

11. What is the average duration of one virtual theory class in your university?

15 minutes

30 minutes

45 minutes

60 minutes

120 minutes

>120 minutes

12. What is the frequency of virtual classes you are attending per week in your university?

One day a week

Two days a week

Three days a week

Four days a week

Five days a week

Six days a week

Seven days a week

Others (please specify):

13. How do you rate the interaction between you and your faculty using virtual learning as compared to classroom learning?

The interaction is better

The interaction is worse

The interaction is the same

14. Which mode of learning in dentistry do you think is more beneficial for you?

Virtual learning

Classroom learning

Both

15. What do you think are the advantages of virtual learning in dentistry? (Multiple responses allowed)

Flexibility of time (can be viewed at a convenient time for the student)

Ease of accessibility (using a computer, smartphone, or tablet)

Comfort of access from any location (no need to be in the classroom)

Affordable (eliminates travel costs and travel time)

Remote access to faculty without geographic limitations

Multidimensional possibility to enhance knowledge through online resources

Ease of assessment (assigned tasks, knowledge, clinical competence)

Environment-friendly

Faster sharing of resources (reading materials, presentations, e-books, videos)

Improve professional networking

Others (please specify):

16. What do you think are the disadvantages of virtual learning in dentistry? (Multiple responses allowed)

Internet connectivity problems

Cost of internet connections

Cost of devices (computer, tablet)

Internet security issues (data, sensitive patient information)

Lack of face-to-face interaction with the faculty

Lack of interaction with patients

Lack of interaction with classmates

Students' weak attitude (laziness, procrastination, etc.)

Need of self-discipline

Poor pre-clinical/clinical/practical orientation

Feeling of isolation

Negative impact on physical and mental health (strain to the eyes, sedentary lifestyle, etc.)

Others (please specify):

17. Would you like ...

conferences/conventions to be held on virtual platforms

paper presentations to be held on virtual platforms

poster presentations to be held on virtual platforms

18. I think virtual learning in dentistry ______________________
